# Supplementary material for: Risk prediction model for post-endoscopic retrograde cholangiopancreatography pancreatitis: A systematic review and meta-analysis
Source: PLoS One. 2025 Sep 15;20(9):e0332378. doi: 10.1371/journal.pone.0332378 (PMC12435719; doi:10.1371/journal.pone.0332378)
Supplement: S2 Table — (DOCX) [file pone.0332378.s002.docx]

**S2 Table.** **PICOTS Framework.**

| **Item** | **Criteria** |
| --- | --- |
| Population | Patients after ERCP |
| Index prediction model | Risk prediction models for pancreatitis after ERCP that were developed and published (predictors ≥ 2) |
| Comparator | No competing model |
| Outcome | The occurrence of pancreatitis post-ERCP |
| Timing | The outcome was predicted after evaluating basic information at admission, comorbidities and past medical history, biomarkers and laboratory values, ERCP procedure-related data, gallbladder and stone condition, duodenal papilla-related data, clinical diagnosis and preventive measures characteristics |
| Setting | The intended use of the risk prediction is to individualize the prediction of pancreatitis in patients undergoing ERCP, facilitating the implementation of preventive measures to prevent adverse events |
