# Supplementary material for: Risk prediction model for post-endoscopic retrograde cholangiopancreatography pancreatitis: A systematic review and meta-analysis
Source: PLoS One. 2025 Sep 15;20(9):e0332378. doi: 10.1371/journal.pone.0332378 (PMC12435719; doi:10.1371/journal.pone.0332378)
Supplement: S3 Table — (DOCX) [file pone.0332378.s003.docx]

**S3 Table.** **Complete List of Search Terms.**

| Search Terms |
| --- |
| (“ERCP”[MESH] OR “Retrograde Cholangiopancreatography, Endoscopic”[Title/Abstract] OR “Cholangiopancreatographies, Endoscopic Retrograde”[Title/Abstract] OR “Endoscopic Retrograde Cholangiopancreatographies”[Title/Abstract] OR “Retrograde Cholangiopancreatographies, Endoscopic”[Title/Abstract] OR “Endoscopic Retrograde Cholangiopancreatography”[Title/Abstract])  AND  (“Pancreatitis”[MESH] OR “Acute Pancreatitis”[Title/Abstract] OR “Acute Pancreatitides”[Title/Abstract] OR “Pancreatitis, Acute Edematous”[Title/Abstract] OR “Acute Edematous Pancreatitides”[Title/Abstract] OR “Peripancreatic Fat Necrosis”[Title/Abstract] OR “Pancreatic Parenchymal Edema”[Title/Abstract])  AND  (“risk assessment”[MESH] OR “risk assessment”[Title/Abstract] OR predict*[Title/Abstract] OR “predictive model”[Title/Abstract] OR “predicting model”[Title/Abstract] OR “risk score”[Title/Abstract] OR “risk model”[Title/Abstract] OR “prognostic model”[Title/Abstract]) OR “risk prediction”[Title/Abstract] OR “risk factors*”[Title/Abstract]) |

MESH, Medical Subject Headings

| **Number** | **Search Terms** | **PubMed** | **Embase** | **Web of Science** | **Cochrane** | **CINAHL** | **CNKI** | **Wanfang** | **VIP** | **SinoMed** | **Google Scholar** | **ClinicalTrials.gov** |
| --- | --- | --- | --- | --- | --- | --- | --- | --- | --- | --- | --- | --- |
| 1 | ((ERCP[MeSH Terms]) OR (Retrograde Cholangiopancreatography, Endoscopic[Title/Abstract]) OR (Cholangiopancreatographies, Endoscopic Retrograde[Title/Abstract]) OR (Endoscopic Retrograde Cholangiopancreatographies[Title/Abstract]) OR (Retrograde Cholangiopancreatographies, Endoscopic[Title/Abstract]) OR (Endoscopic Retrograde Cholangiopancreatography[Title/Abstract])) | 28,047 | 34,781 | 53,402 | 3,086 | 10,381 | 12,734 | 34,213 | 12,257 | 19,421 | 547,291 | 499 |
| 2 | ((Pancreatitis[MeSH Terms]) OR (Acute Pancreatitis[Title/Abstract]) OR (Acute Pancreatitides[Title/Abstract]) OR (Pancreatitis, Acute Edematous[Title/Abstract]) OR (Acute Edematous Pancreatitides[Title/Abstract]) OR (Peripancreatic Fat Necrosis) OR (Pancreatic Parenchymal Edema)) | 442,682 | 159,036 | 108,592 | 17,333 | 26,725 | 70,474 | 138,752 | 68,835 | 50,657 | 1,003,605 | 800 |
| 3 | ((risk assessment”[MESH Terms] OR (risk assessment[Title/Abstract]) OR predict*[Title/Abstract] OR (predictive model[Title/Abstract]) OR (predicting model[Title/Abstract]) OR (risk score[Title/Abstract]) OR (risk model[Title/Abstract]) OR (prognostic model[Title/Abstract]) OR (risk prediction[Title/Abstract]) OR (risk factor*[Title/Abstract]) OR nomogram*[Title/Abstract])) | 4,707,306 | 181,345 | 501,904 | 264,501 | 5,843 | 366,358 | 90,634 | 433,438 | 5,268 | 315,438 | 3,218 |
| 4 | 1 AND 2 AND 3 | 2,837 | 2,297 | 1,637 | 332 | 190 | 51 | 38 | 17 | 139 | 527 | 1 |

Total = 8,066

After removal of duplicates (Endnote) = 4,301

Full text review = 24
