# Supplementary material for: Risk prediction model for post-endoscopic retrograde cholangiopancreatography pancreatitis: A systematic review and meta-analysis
Source: PLoS One. 2025 Sep 15;20(9):e0332378. doi: 10.1371/journal.pone.0332378 (PMC12435719; doi:10.1371/journal.pone.0332378)
Supplement: S4 Table — (DOCX) [file pone.0332378.s004.docx]

**S4 Table.** **Risk of bias assessment using the Newcastle Ottawa Scale.**

| **Study** | **Selection** | **Comparability** | **Exposure** | **Total** |
| --- | --- | --- | --- | --- |
| Friedland 2002 | 4 | 1 | 3 | 8 |
| Dimagno 2013 | 4 | 1 | 2 | 7 |
| Fang 2016 | 3 | 1 | 3 | 7 |
| Wan 2018 | 3 | 1 | 3 | 7 |
| Chiba 2021 | 3 | 2 | 3 | 8 |
| Dou 2021 | 3 | 1 | 3 | 7 |
| Wang 2021 | 3 | 1 | 3 | 7 |
| Zhang 2021 | 3 | 1 | 3 | 7 |
| Zheng 2021 | 3 | 2 | 3 | 8 |
| Park 2021 | 3 | 1 | 3 | 7 |
| Fujita 2021 | 3 | 1 | 3 | 7 |
| Zhang 2022 | 3 | 2 | 3 | 8 |
| Fu 2022 | 3 | 1 | 3 | 7 |
| Huang 2022 | 3 | 2 | 3 | 8 |
| Archibugi 2022 | 4 | 1 | 3 | 8 |
| Ma 2023 | 3 | 1 | 3 | 7 |
| Yao 2023 | 3 | 2 | 3 | 8 |
| Qin 2023 | 3 | 2 | 3 | 8 |
| Wang 2023 | 3 | 2 | 3 | 8 |
| Chen 2023 | 3 | 1 | 3 | 7 |
| Takahashi 2023 | 3 | 1 | 3 | 7 |
| Fukuda 2023 | 3 | 1 | 3 | 7 |
| Zhang 2023 | 3 | 1 | 3 | 7 |
| Yan 2024 | 4 | 2 | 3 | 9 |
