# Supplementary material for: Risk prediction model for post-endoscopic retrograde cholangiopancreatography pancreatitis: A systematic review and meta-analysis
Source: PLoS One. 2025 Sep 15;20(9):e0332378. doi: 10.1371/journal.pone.0332378 (PMC12435719; doi:10.1371/journal.pone.0332378)
Supplement: S5 Table — (DOCX) [file pone.0332378.s005.docx]

**S5 Table. GRADE assessment for post-endoscopic retrograde cholangiopancreatography pancreatitis.**

| **№ of studies** | **Certainty assessment** | | | | | | **Effect** | | | **Certainty** | **Importance** |
| --- | --- | --- | --- | --- | --- | --- | --- | --- | --- | --- | --- |
|  | **Study design** | **Risk of bias** | **Inconsistency** | **Indirectness** | **Imprecision** | **Publication bias** | **№ of events** | **№ of individuals** | **Rate (95% CI)** |  |  |
| cholangiopancreatography pancreatitis | | | | | | | | | | | |
| 24 | non-randomised studies | serious | serious^a^ | not serious | not serious | not serious | 2623 | 38016 | event rate 8.8% (6.57 to 10.93) | ⨁⨁⨁〇  Moderate | IMPORTANT |

#### Explanations

a. The high statistical heterogeneity (I² = 84%) in model discrimination suggests significant between-study variability, likely reflecting differences in endoscopist proficiency. Consequently, we applied a one-level downgrade for inconsistency.
