# Supplementary material for: Risk prediction model for post-endoscopic retrograde cholangiopancreatography pancreatitis: A systematic review and meta-analysis
Source: PLoS One. 2025 Sep 15;20(9):e0332378. doi: 10.1371/journal.pone.0332378 (PMC12435719; doi:10.1371/journal.pone.0332378)
Supplement: S1 Fig — (DOCX) [file pone.0332378.s006.docx]

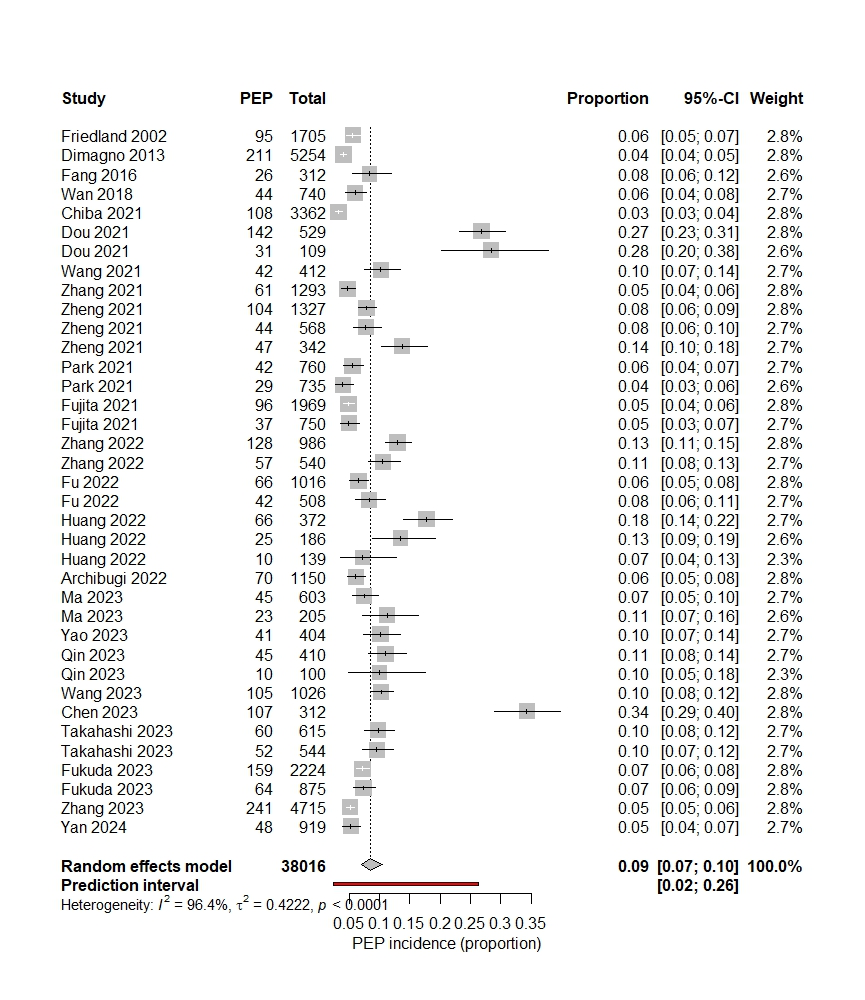


**S1 Fig. Forest plot of Post-ERCP Pancreatitis incidence: a meta-analysis.**

The forest plot presents a meta-analysis of PEP incidence across 24 studies (Friedland 2002 to Yan 2024), comprising 38,016 total procedures. Random-effects model revealed a pooled PEP incidence of 9% (95% CI: 7%-10%). Prediction interval ranged widely from 2% to 26%, indicating substantial heterogeneity. I² = 96.4% ( p < 0.0001), τ² = 0.4222, confirming high between-study variability. Individual study proportions varied from 3% (Chiba 2021) to 34% (Chen 2023). Studies were weighted 2.3%-2.8%, with larger studies (e.g., Zhang 2023, n =4,715) contributing proportionally to precision.
