# Supplementary material for: Risk prediction model for post-endoscopic retrograde cholangiopancreatography pancreatitis: A systematic review and meta-analysis
Source: PLoS One. 2025 Sep 15;20(9):e0332378. doi: 10.1371/journal.pone.0332378 (PMC12435719; doi:10.1371/journal.pone.0332378)
Supplement: S2 Fig — (DOCX) [file pone.0332378.s007.docx]

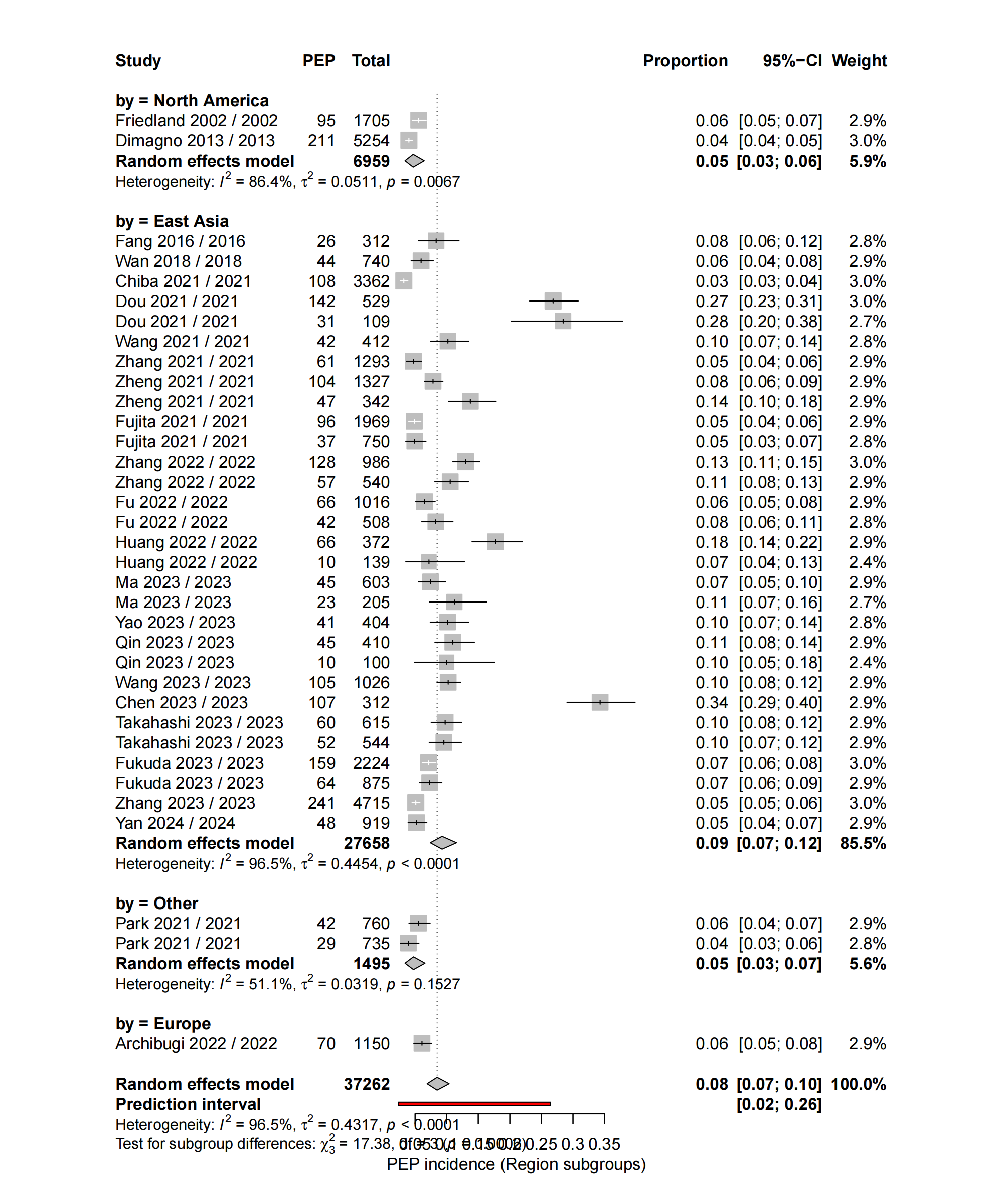


**S2 Fig. Forest plot of Post-ERCP Pancreatitis incidence: subgroup meta-analysis by geographic region.**

Across 24 studies (N=37,262 procedures), the overall PEP incidence was 8% (95% CI: 7%-10%) (random-effects model). Prediction interval: 2%-26%, indicating substantial heterogeneity (I²=96.5%, τ²=0.4317, p <0.0001). Significant subgroup differences (χ²=17.36, p =0.008). East Asia showed the highest incidence and heterogeneity (τ²=0.445 vs. 0.051 in North America).
