# Supplementary material for: Risk prediction model for post-endoscopic retrograde cholangiopancreatography pancreatitis: A systematic review and meta-analysis
Source: PLoS One. 2025 Sep 15;20(9):e0332378. doi: 10.1371/journal.pone.0332378 (PMC12435719; doi:10.1371/journal.pone.0332378)
Supplement: S3 Fig — (DOCX) [file pone.0332378.s008.docx]

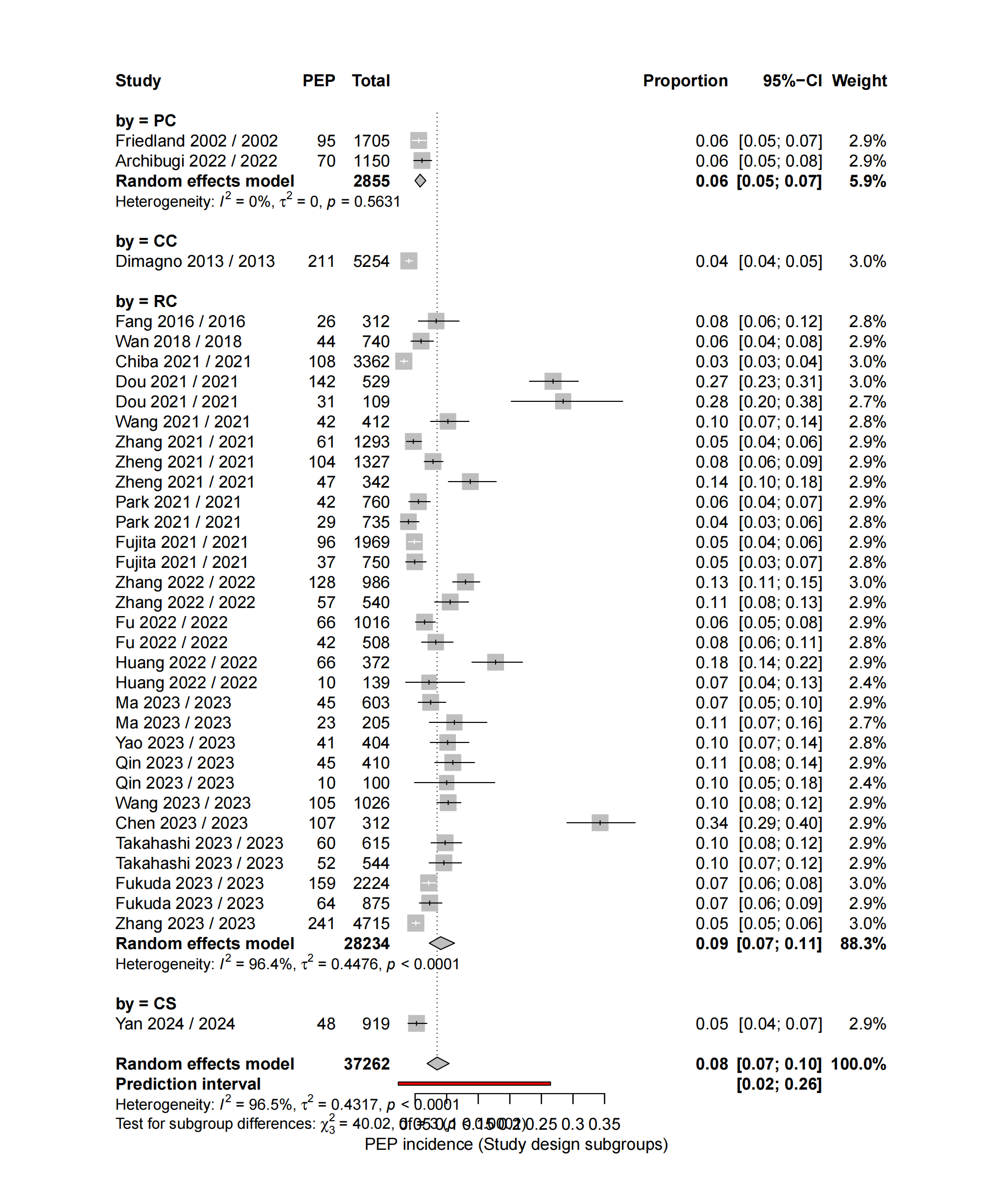


**S3 Fig. Forest plot of Post-ERCP Pancreatitis incidence: subgroup meta-analysis by study design.**

Random-effects model incorporating 24 studies (N=37,262 procedures) demonstrated a pooled PEP incidence of 8% (95% CI: 7%-10%). Prediction interval ranged from 2% to 26%, indicating substantial heterogeneity (I²=96.5%, τ²=0.4317, p <0.0001). Significant subgroup differences (χ²=40.02, p <0.001). RC studies showed highest variability (I²=96.4%, τ²=0.4476).
