# Supplementary material for: Risk prediction model for post-endoscopic retrograde cholangiopancreatography pancreatitis: A systematic review and meta-analysis
Source: PLoS One. 2025 Sep 15;20(9):e0332378. doi: 10.1371/journal.pone.0332378 (PMC12435719; doi:10.1371/journal.pone.0332378)
Supplement: S4 Fig — (DOCX) [file pone.0332378.s009.docx]

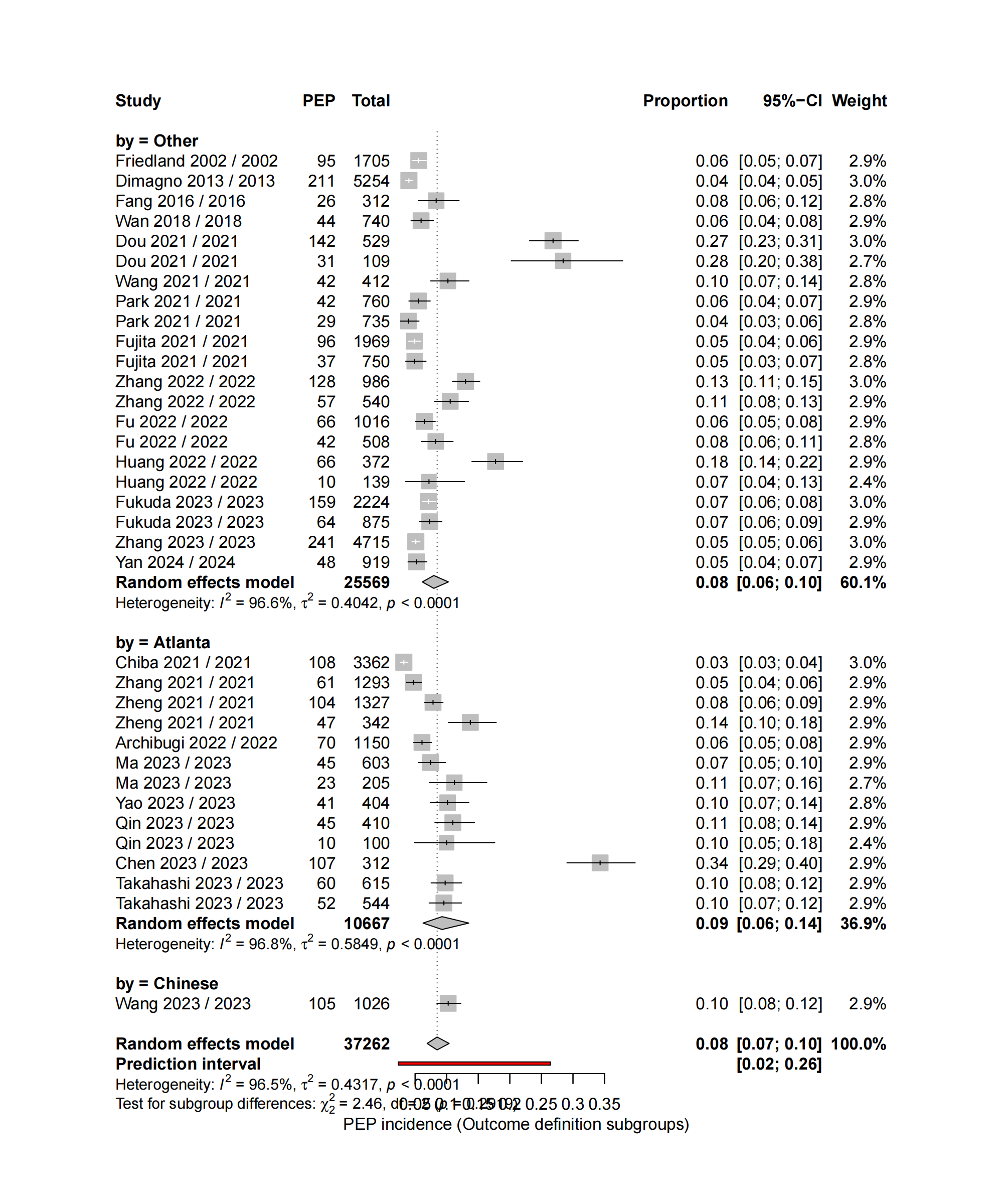


**S4 Fig. Forest plot of Post-ERCP Pancreatitis incidence: subgroup meta-analysis by diagnostic criteria.**

Across 24 studies (N=37,262 procedures), the random-effects model estimated a PEP incidence of 8% (95% CI: 7%-10%). Prediction interval spanned 2%-26%, reflecting extreme heterogeneity (I²=96.5%, τ²=0.4317, p <0.0001). No significant subgroup differences (χ²=2.46, p =0.29). Atlanta-criteria studies exhibited wider CIs and higher between-study variance (τ²=0.585 vs. 0.404).
