# Supplementary material for: Risk prediction model for post-endoscopic retrograde cholangiopancreatography pancreatitis: A systematic review and meta-analysis
Source: PLoS One. 2025 Sep 15;20(9):e0332378. doi: 10.1371/journal.pone.0332378 (PMC12435719; doi:10.1371/journal.pone.0332378)
Supplement: S6 Fig — (DOCX) [file pone.0332378.s011.docx]

A


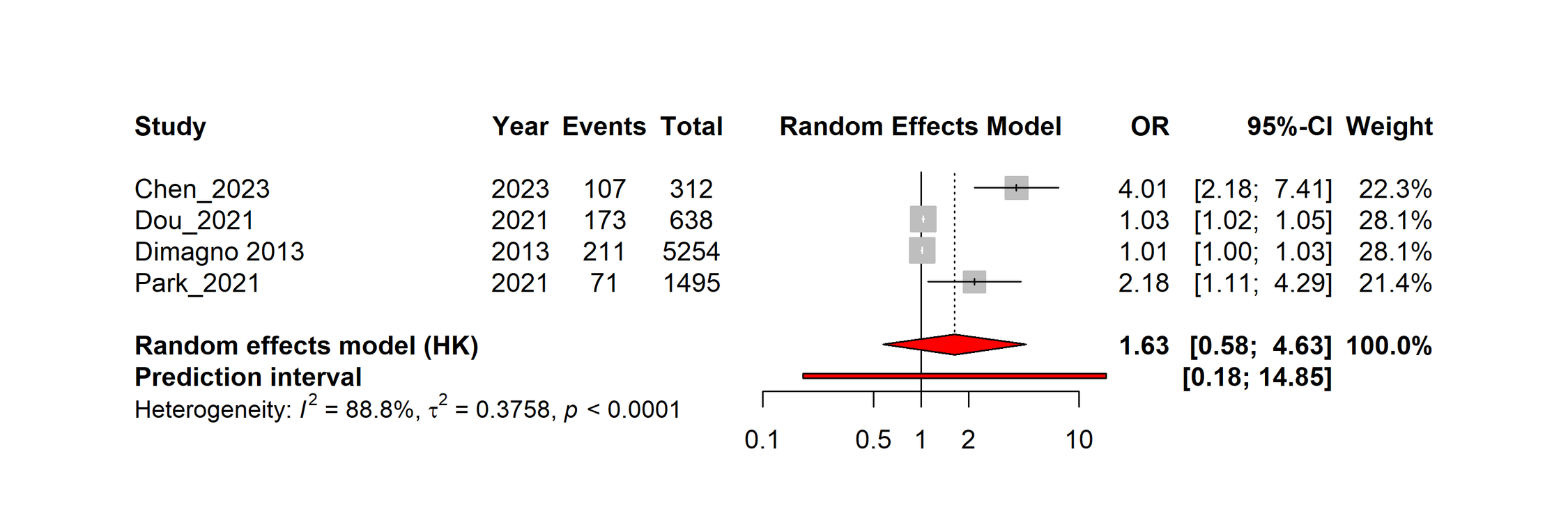


B


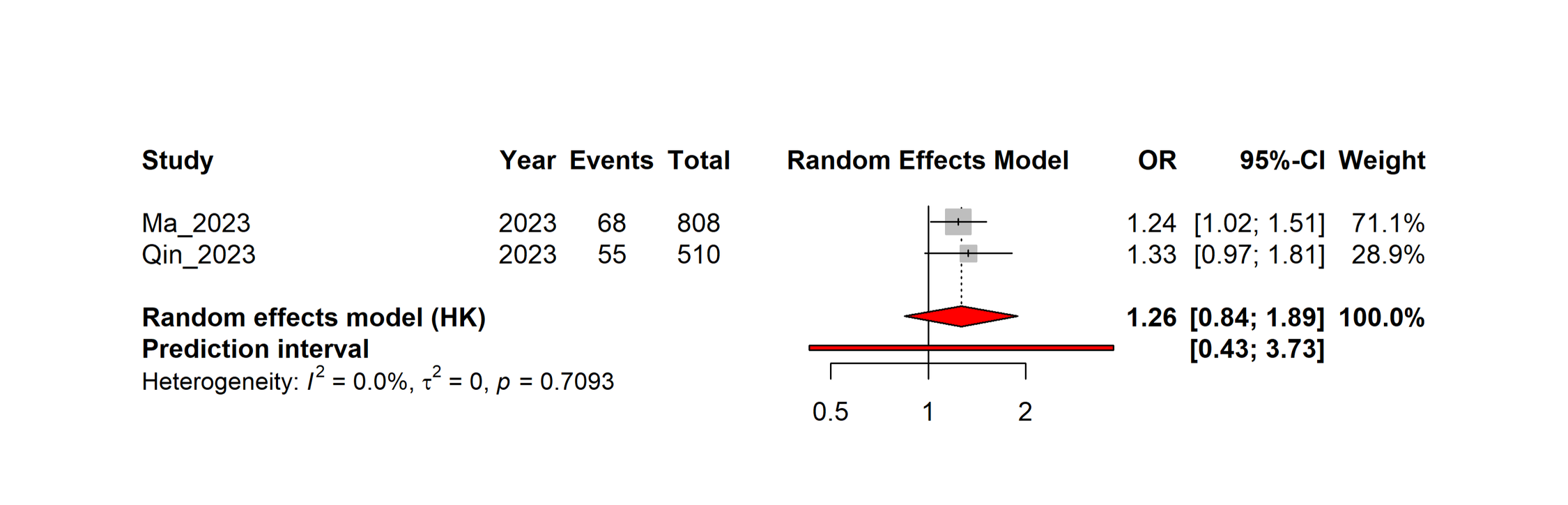


C


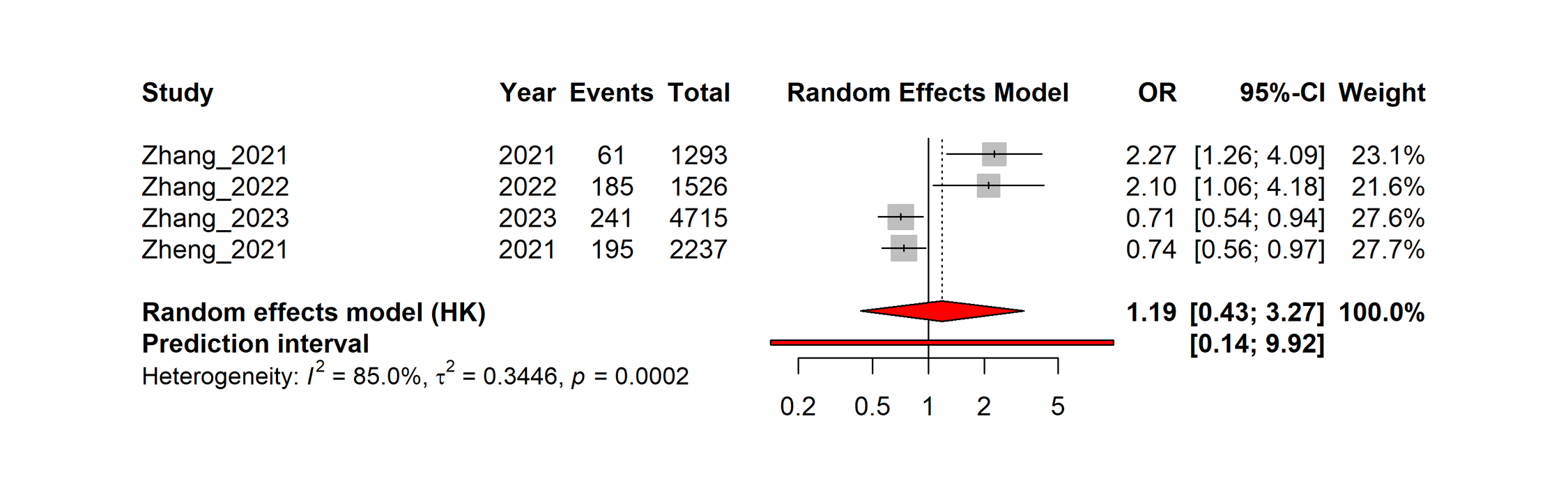


D


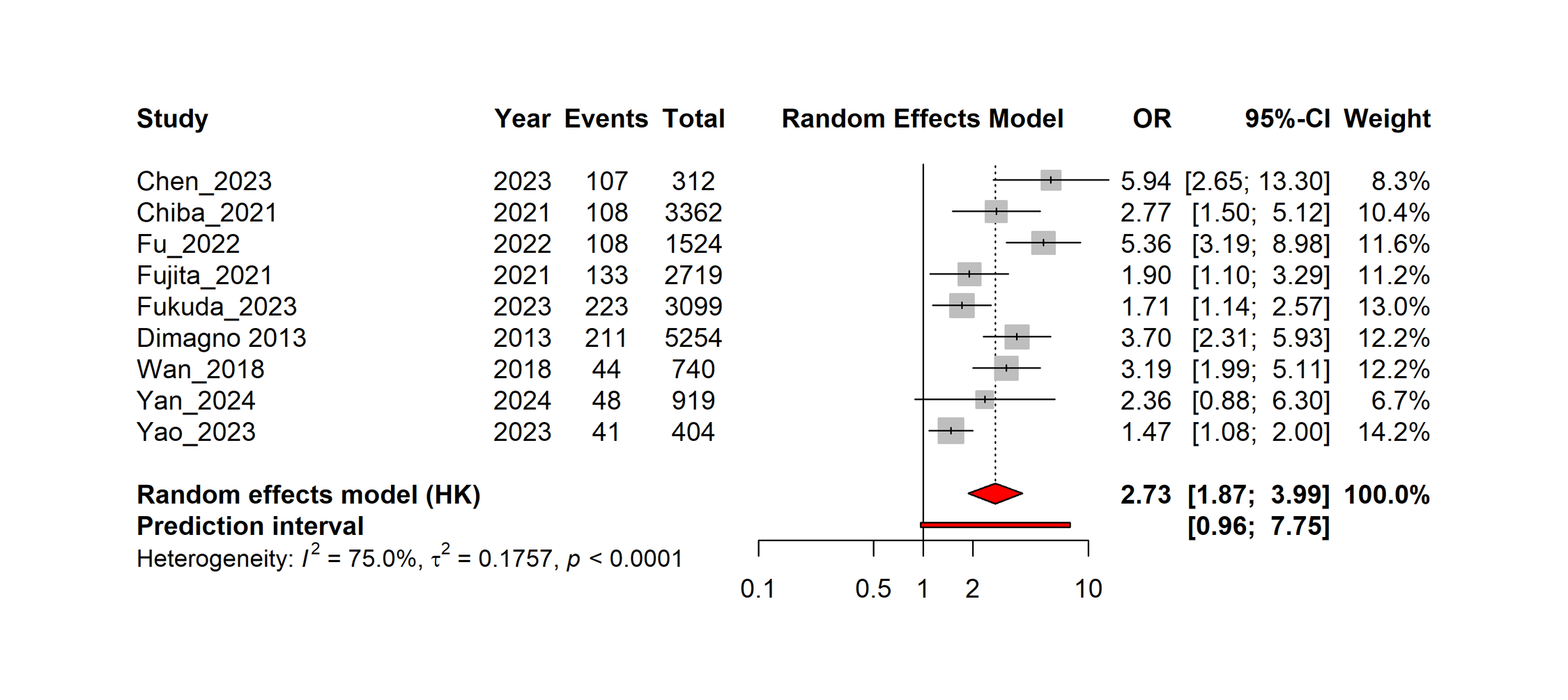


E


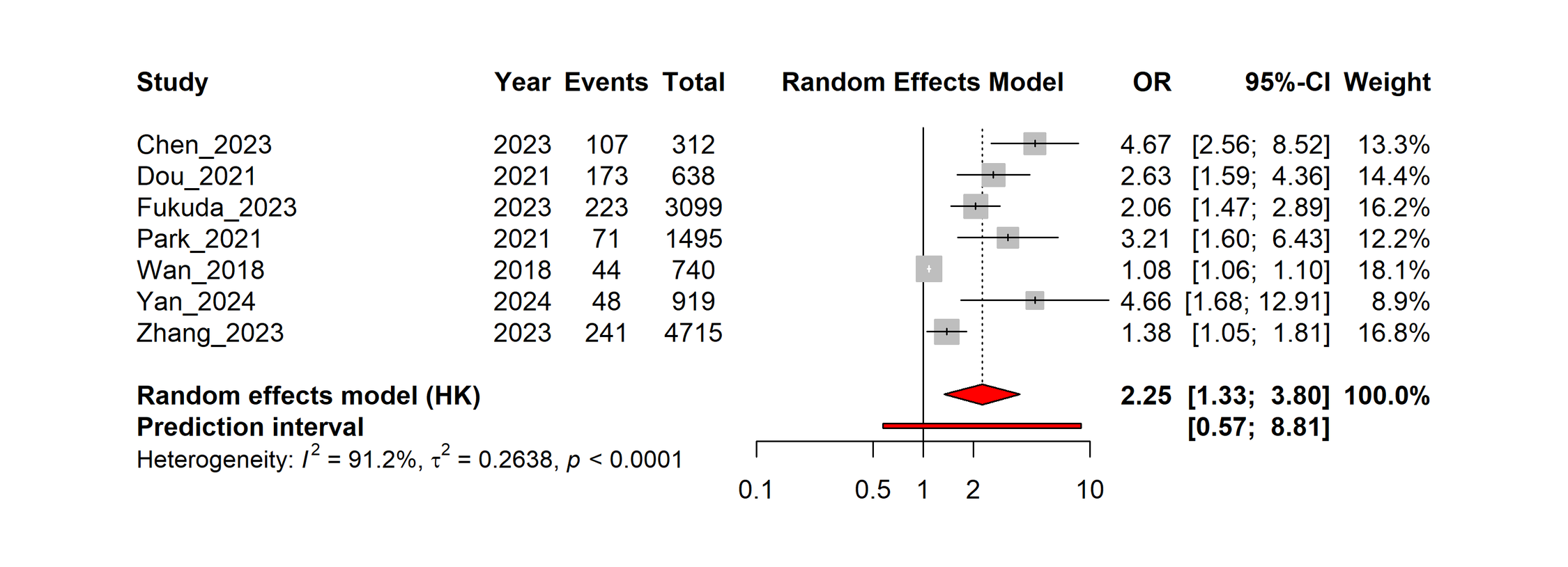


F


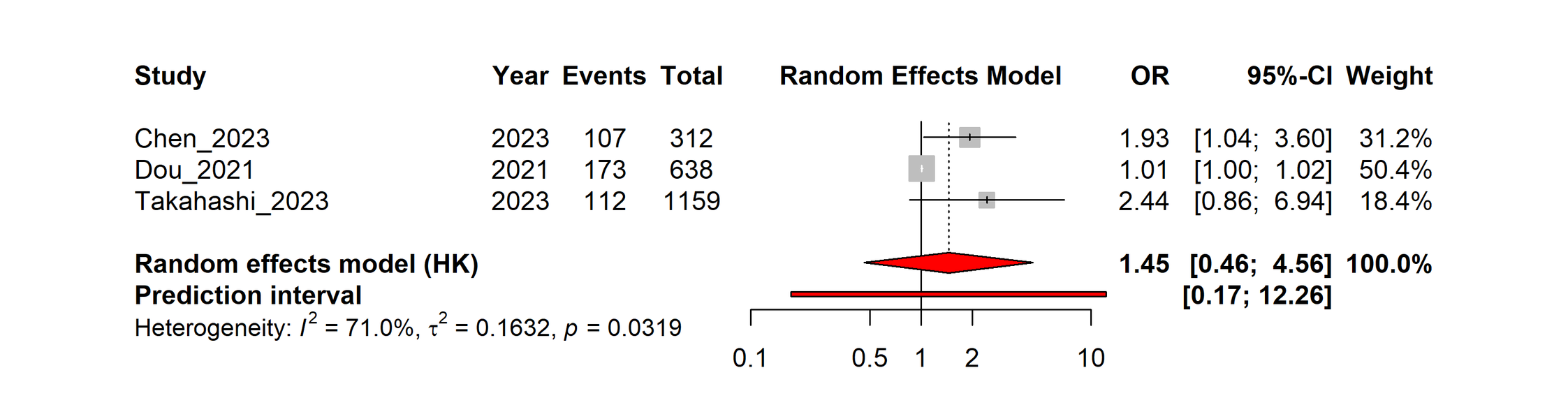


G


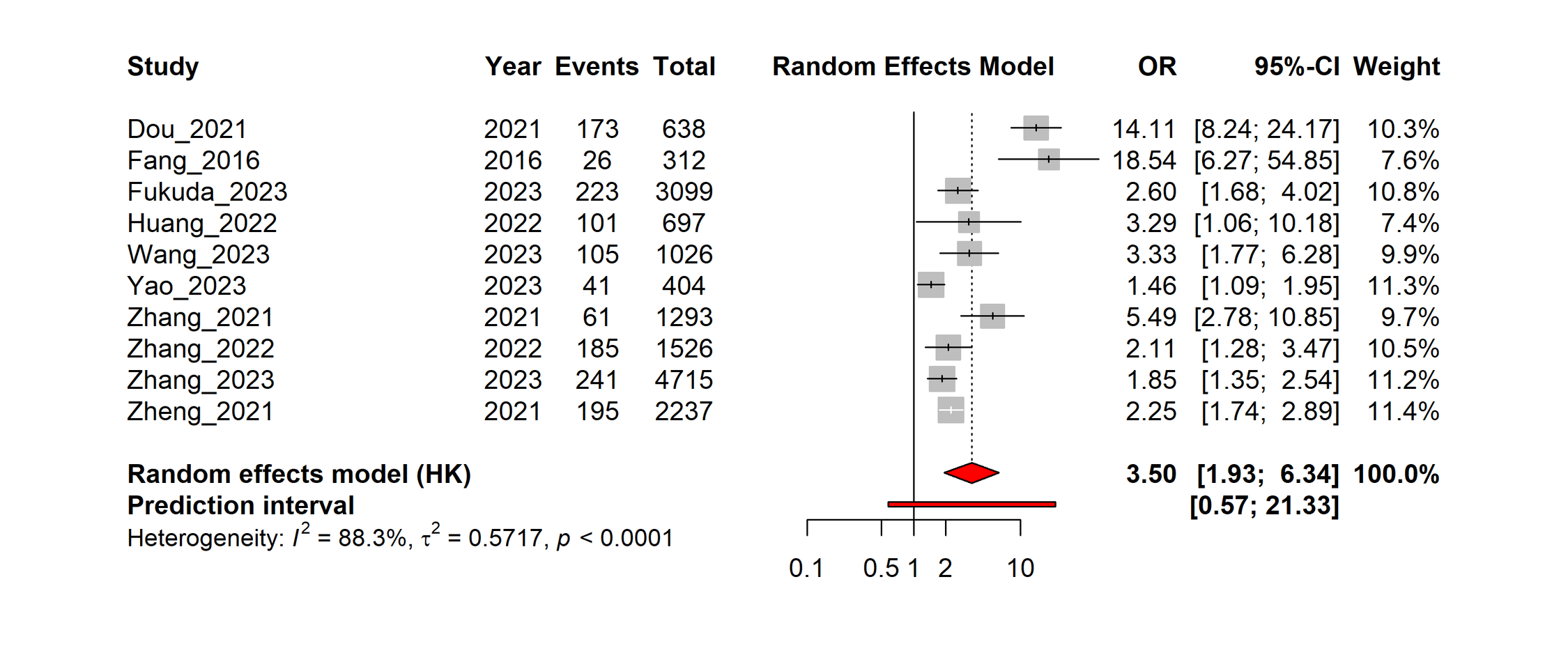


H


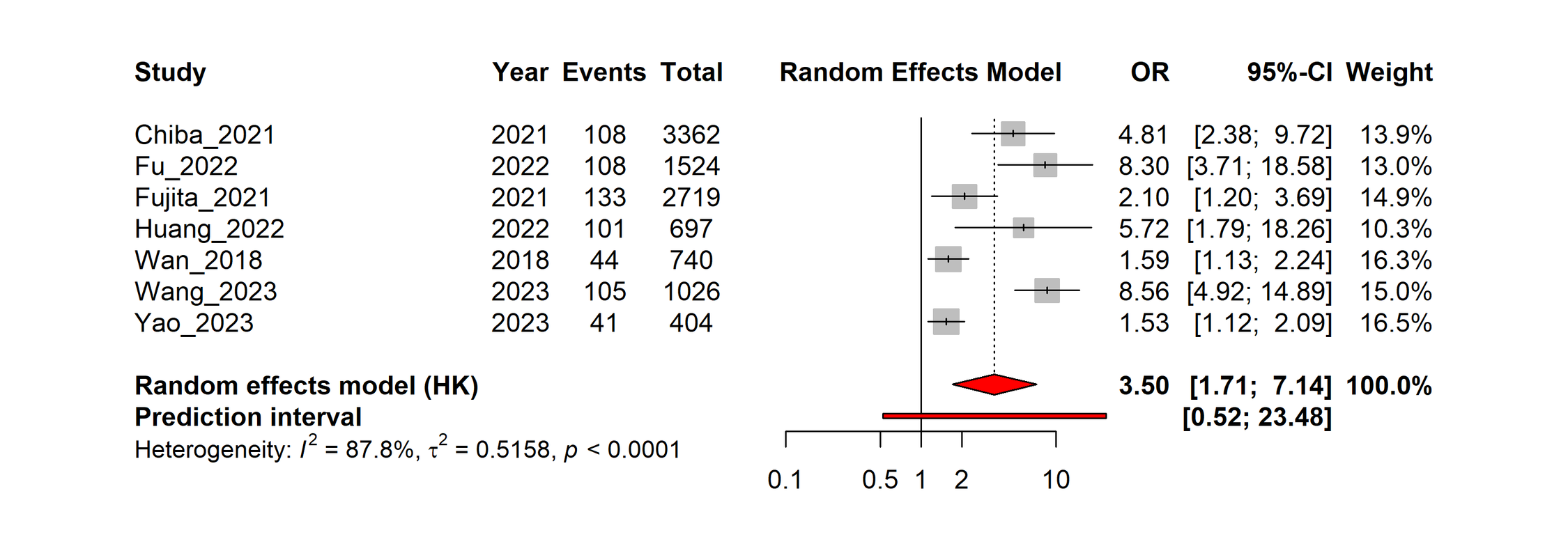


I


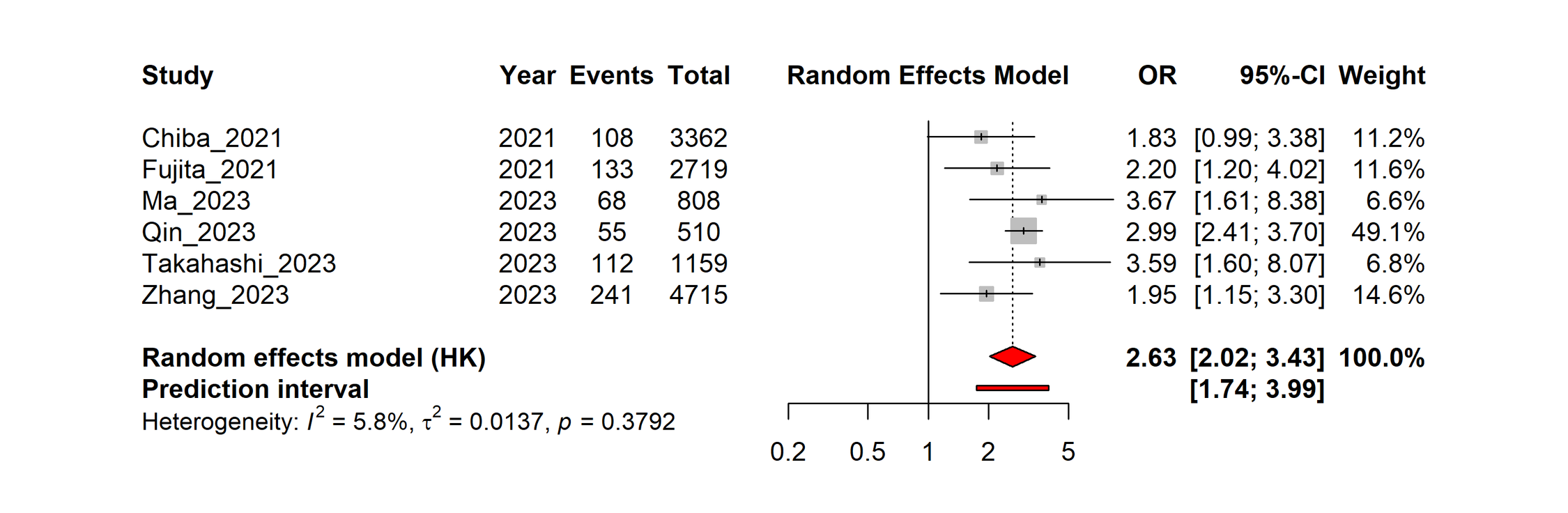


J


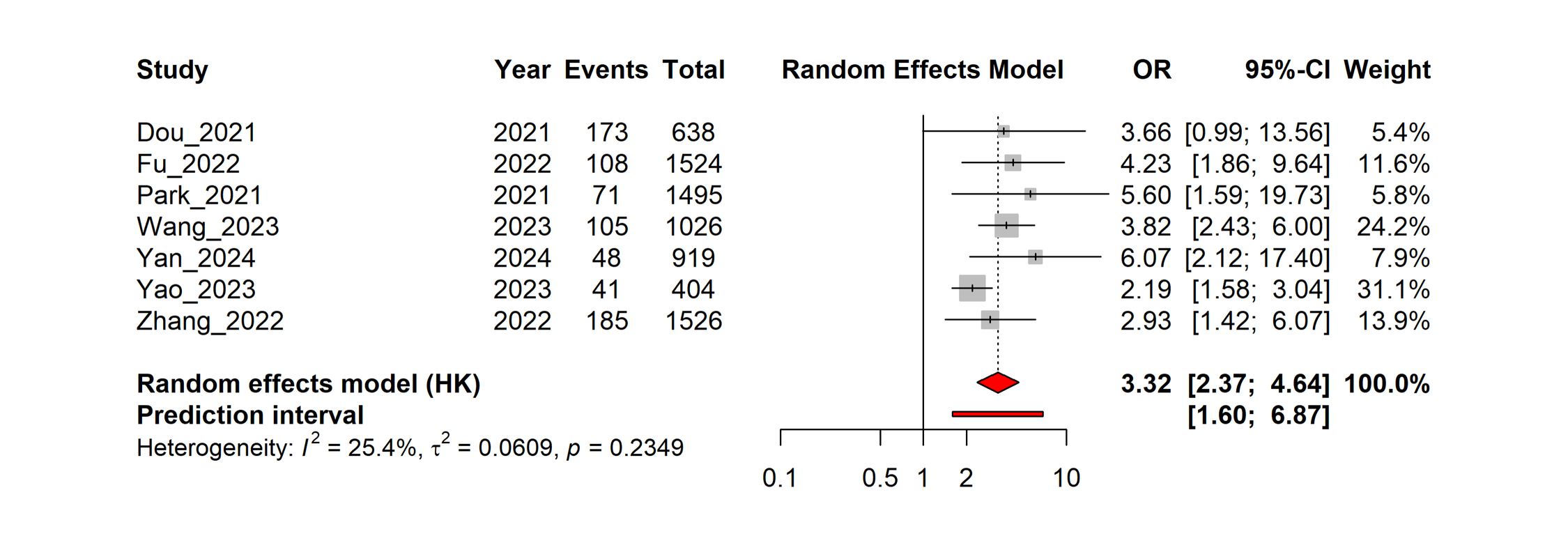


K


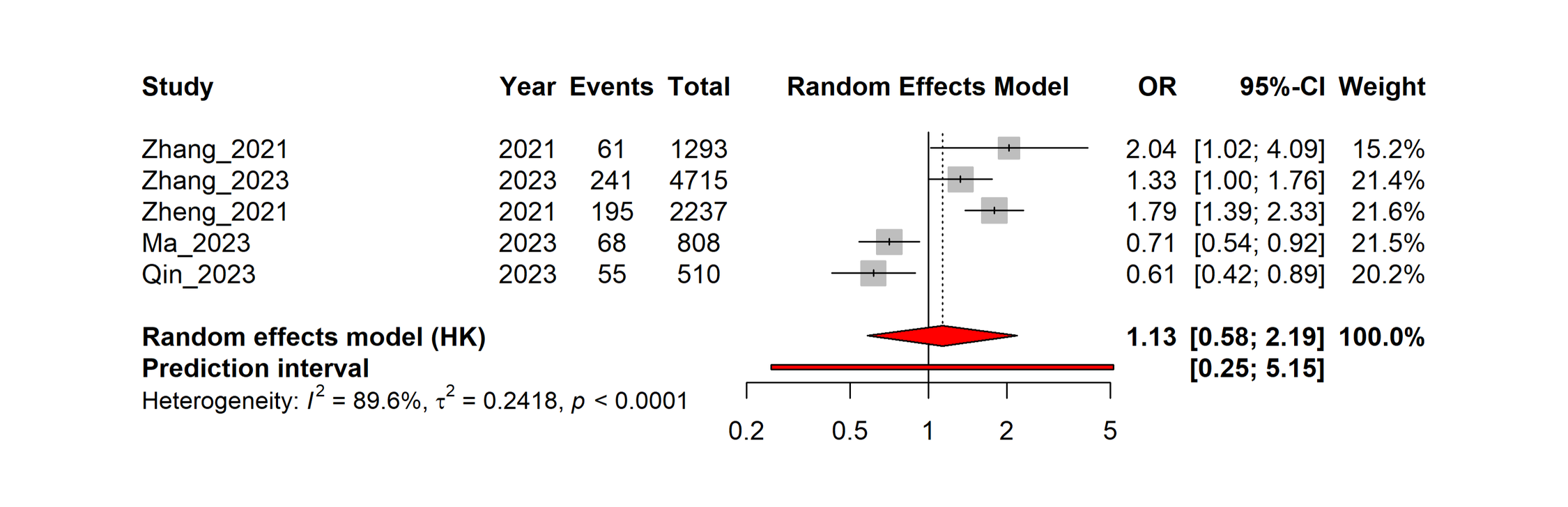


**S6 Fig. Forest plot of the meta-analysis on risk factors for Post-ERCP Pancreatitis.**

(A)Forest plot for age. (B) Forest plot for ALB. (C) Forest plot for calculus of common bile duct. (D) Forest plot for difficult cannulation. (E) Forest plot for female gender. (F) Forest plot for operation time.(G) Forest plot for pancreatic duct cannulation. (H) Forest plot for pancreatic injection. (I) Forest plot for PGW. (J) Forest plot for previous pancreatitis. (K) Forest plot for TBIL.
