# Supplementary material for: Risk prediction model for post-endoscopic retrograde cholangiopancreatography pancreatitis: A systematic review and meta-analysis
Source: PLoS One. 2025 Sep 15;20(9):e0332378. doi: 10.1371/journal.pone.0332378 (PMC12435719; doi:10.1371/journal.pone.0332378)
Supplement: S7 Fig — (DOCX) [file pone.0332378.s012.docx]

A


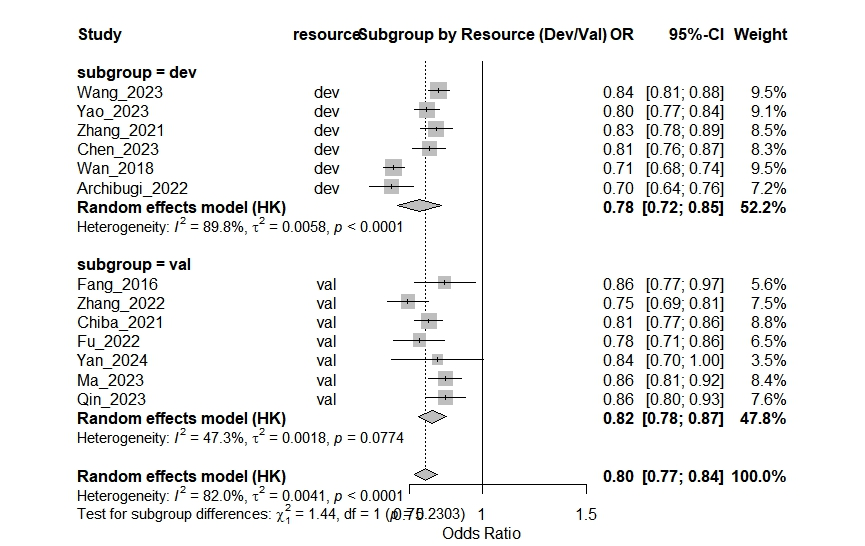


B


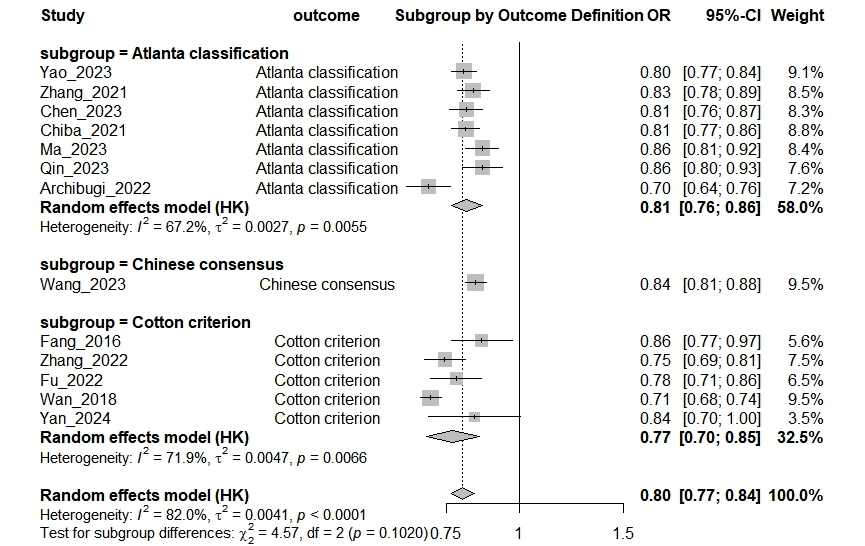


C


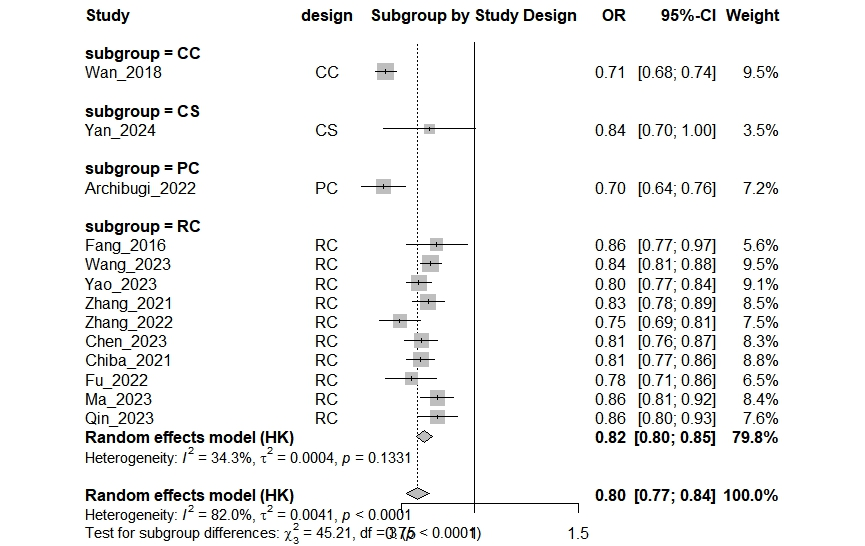


D


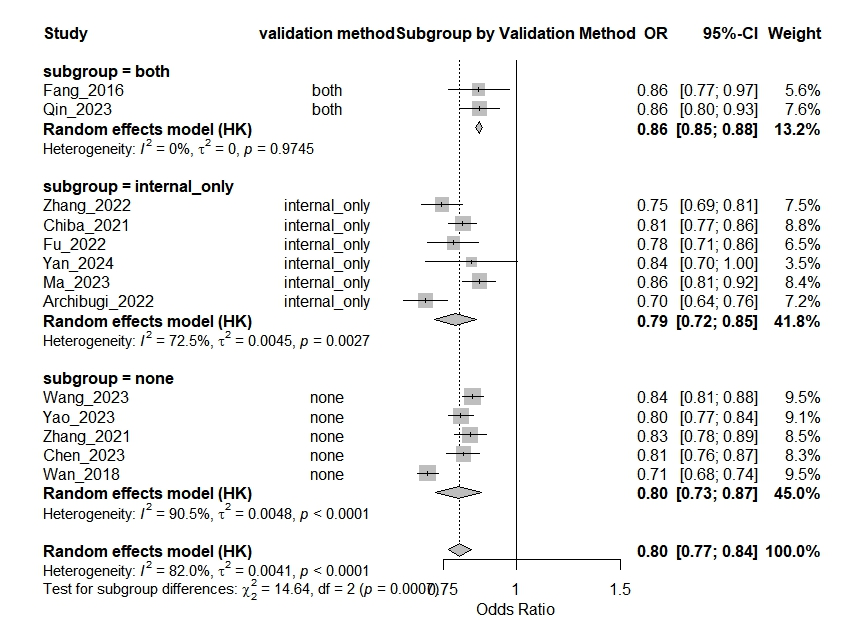


E


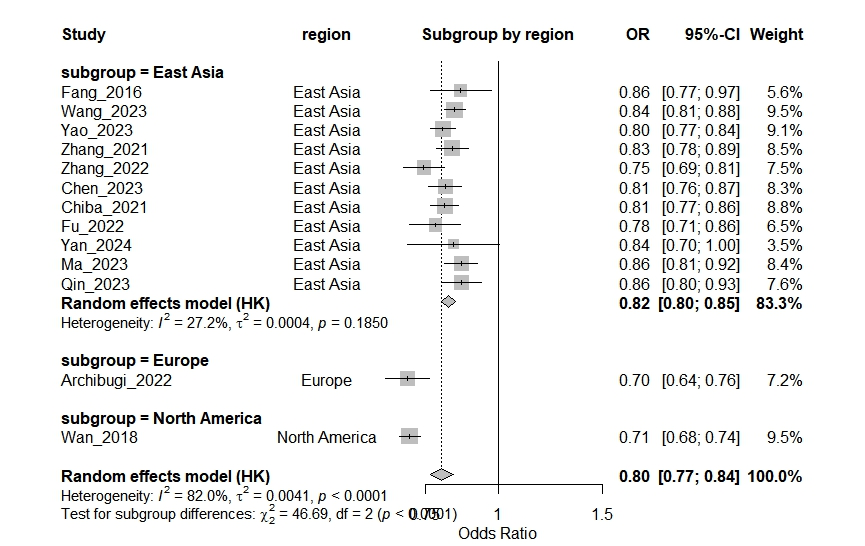


F


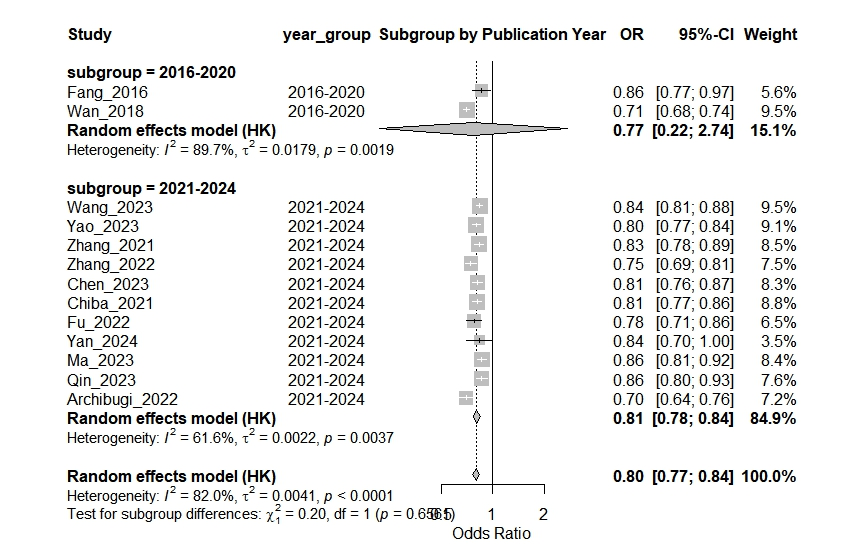


G


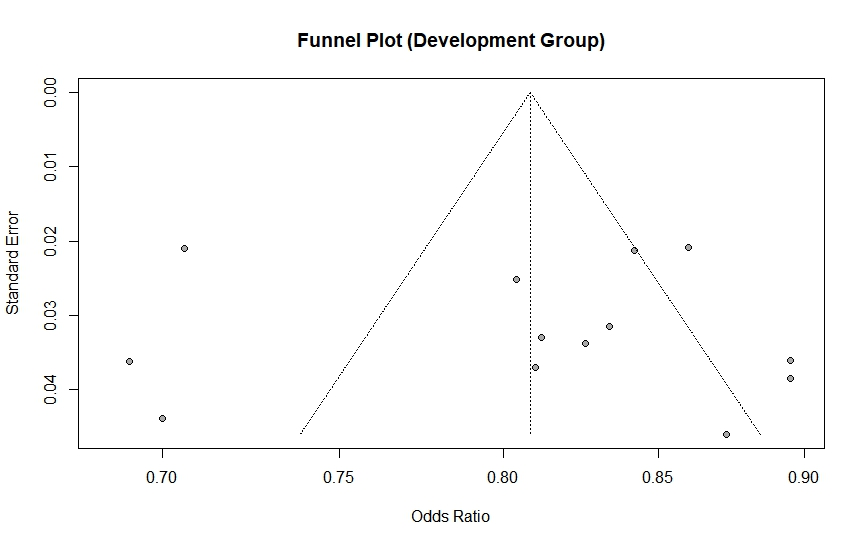


H


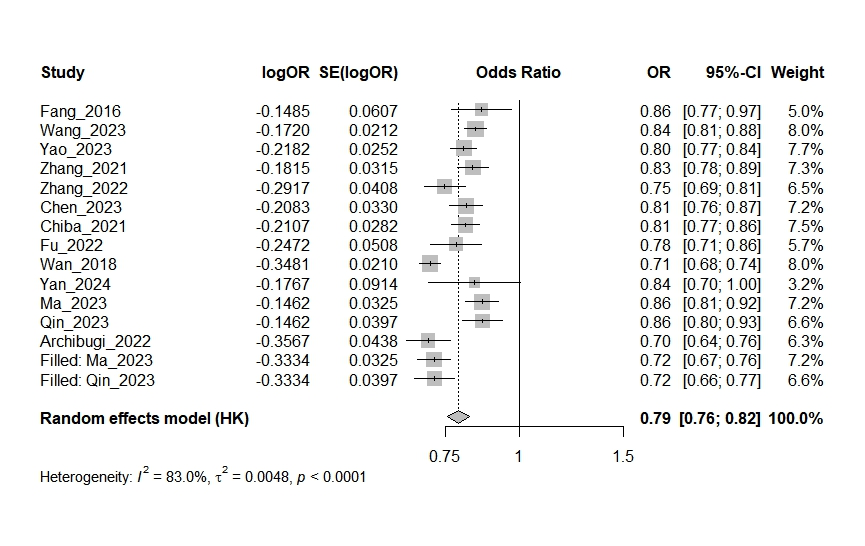


**S7 Fig. Forest plot of the meta-analysis on predictive performance for Post-ERCP Pancreatitis.**

(A)Forest plot for dataset type. (B) Forest plot for diagnostic criteria. (C) Forest plot for study design. (D) Forest plot for validation method. (E) Forest plot for geographic region. (F) Forest plot for publication year. (G) Funnel plot. (H) Sensitivity analysis.
