# Supplementary material for: Risk prediction model for post-endoscopic retrograde cholangiopancreatography pancreatitis: A systematic review and meta-analysis
Source: PLoS One. 2025 Sep 15;20(9):e0332378. doi: 10.1371/journal.pone.0332378 (PMC12435719; doi:10.1371/journal.pone.0332378)
Supplement: Attachment — Submitted filename: Response to Reviewers.doc [file pone.0332378.s014.doc]

Dear Dr. Suphakarn Techapongsatorn,

Thank you for giving us the opportunity to revise our manuscript [ Manuscript ID: PONE-D-24-50343, Title: Risk prediction model for post-endoscopic retrograde cholangiopancreatography pancreatitis: A systematic review and meta-analysis ]. We sincerely appreciate the reviewers’ constructive comments, which have helped us significantly improve the paper.

We have carefully addressed all comments point-by-point in this response letter. Major revisions are highlighted in **yellow** in the manuscript.

We believe the revised manuscript now meets the journal’s standards. Please find our detailed responses below.

Sincerely,

Yijun Mao, Qiang Liu, Hui Fan, Wenjing He, Cheng Zhang, Xueqian Ouyang, Erqing Li, Xiaojuan Wang, Li Qiu, Huanni Dong

Xianyang Central Hospital

[hlb33288602@163.com](mailto:hlb33288602@163.com)

445829791@qq.com

**Reviewer #1:**

We thank the reviewer for their insightful suggestions.

**Comment 1:** The inclusion criteria could be optimized. The included studies are only observational, which limits the diversity of the model sources. It is advisable to consider incorporating some interventional studies to comprehensively evaluate the performance of the model.

**Response:**

We sincerely appreciate this constructive suggestion. In response:

**1. Criteria Modification:**

We have revised the inclusion criteria to explicitly allow for interventional studies containing PEP prediction models (Section **Inclusion and exclusion criteria**, Page 6). This change enhances the scope while maintaining our focus on model validation.

**2. Current Study Rationale:**

Our initial restriction to observational studies aimed to ensure homogeneity in model derivation populations, as most ERCP prediction models are developed using observational data.[1-3]

**3. Future Direction:**

We fully agree that interventional studies could provide valuable insights into model performance under controlled conditions. This limitation is now acknowledged in the Discussion (Page 24, Lines 496-499).

**Changes made:**

- Inclusion criteria updated in Methods

- Limitations section expanded

- Added references supporting observational data predominance in model development

**Comment 2:** Insufficient consideration of multi - factor interactions: When exploring predictive factors, only the relationship between a single factor and PEP was analyzed, without considering the interactions among factors. It is recommended to supplement relevant analyses to more accurately reveal the disease risk mechanisms.

**Response:**

We sincerely appreciate this insightful suggestion. We have now supplemented the analysis of multifactor interactions as follows:

**1. Mechanistic discussion:**

Added a dedicated subsection (Page 20-21) explaining how factor interactions may synergistically increase PEP risk through:

• Mechanical trauma compounding (e.g., repeated cannulation + guidewire)

• Physiological susceptibility modulation (e.g., gender-specific sphincter reactivity)

• Metabolic stress amplification (e.g., hypoalbuminemia + prolonged ischemia)

**Comment 3:** The search strategy can be refined. The search terms only involve common keywords, which may lead to the omission of relevant literature. It is recommended to supplement synonyms and abbreviations and search more databases, such as ClinicalTrials.gov and Google Scholar, to ensure comprehensiveness.

**Response:**

We sincerely appreciate the reviewer’s constructive suggestions to improve the literature search comprehensiveness.

**1. Database expansion:**

Added **ClinicalTrials.gov** and **Google Scholar** per the reviewer’s suggestion.

**2. Validation:**

Revised search strategy yielded no additional eligible studies.

**3.Supplemented the detailed retrieval strategies for all databases in:**

S2 Table. PICOTS Framework

S3 Table. Complete List of Search Terms

**4.Added the following elements to ensure reproducibility:**

Complete search syntax with Boolean operators

Both controlled vocabulary (e.g., MeSH) and free-text terms

Number of records retrieved from each database

**5.Cited these supplementary tables in the Methods section (subsection "Search strategy", Page 5, Line 107).**

**Changes made:**

S2 Table and S3Table: Provided complete search syntax for all databases.

We confirm these modifications have significantly enhanced the study’s methodological rigor.

**Comment 4:** Limitations of the quality assessment tool: Only the PROBAST tool is used to assess the risk of bias. It would be beneficial to incorporate other tools, such as the Jadad quality scoring scale, to more accurately evaluate the quality of studies from multiple dimensions.

**Response:**

We sincerely appreciate this constructive suggestion. To address the concern about multidimensional quality evaluation, we have implemented the following improvements:

1. **Methodological Enhancement**

Added Newcastle-Ottawa Scale (NOS) for observational studies alongside PROBAST

This provides complementary perspectives:
✓ PROBAST: Model-specific risk of bias
✓ NOS: General study quality metrics

2. **Results Expansion**

Created S4 Table comparing quality ratings across studies (Page 13, Lines 274-277)

3. **Transparent Reporting**

Clearly labeled the secondary role of NOS in manuscript (Page 7, Lines 141-143)

**Changes made:**

Methods: Page 7, Section **Quality assessment**

Results: Page 13, Lines 274-277, New S4 Table

We believe these modifications have strengthened the methodological rigor while maintaining focus on prediction model-specific assessment.

**Comment 5:** Analysis of model performance indicators is lacking: When reporting model performance, the analysis of the C - statistic and calibration results is rather superficial. It is necessary to delve deeper into the reasons for the performance differences among different models and the impact of these differences on clinical applications.

**Response:**

We sincerely appreciate this constructive suggestion. We have significantly expanded the analysis of model performance in the revised manuscript:

1. Stratified analysis of C-statistic performance with high/low performance model comparisons.
2. Detailed examination of predictor categories contributing to discrimination differences
3. Calibration assessment with both statistical tests and graphical analysis
4. Clinical applicability discussion based on model complexity

**Changes made:**

Revised text in Section **Model performance** (Page 10-11).

**Comment 6:** Currently, the paper has not conducted GRADE grading on the quality of evidence. It is recommended that, based on the GRADE system, the included studies be comprehensively evaluated in terms of research design, risk of bias, inconsistency, indirectness, publication bias, etc., and the quality grades of each study and the overall evidence be provided. Through GRADE grading, readers can more intuitively understand the reliability of the evidence, providing clearer guidance for subsequent clinical decision-making and further research.

**Response:**

We sincerely appreciate this constructive suggestion. Following the GRADE methodology, we have:

1. Added a dedicated evaluation section in Methods (Page 7, Lines 143-146)
2. Created S5 Table summarizing quality assessment for all outcomes
3. The GRADE classification outcomes and a detailed analysis of each dimension in Results (Page 13-14)

**Key Findings:**
The overall evidence quality was rated as moderate due to:

- Partial studies failed to report model calibration details, resulting in an overall moderate risk of bias (downgraded for risk of bias)
- Substantial heterogeneity in model discrimination was observed (I²=84%, downgraded for inconsistency)

**Changes made:**

Methods: Page 7, Lines 143-146

Results: Page 13-14, New S5 Table

**Comment 7:** Please supplement the detailed retrieval strategies for each database to enhance the scientific nature and reproducibility of the research.

**Response:**

1. **Supplemented the detailed retrieval strategies for all databases in:**

S2 Table. PICOTS Framework

S3 Table. Complete List of Search Terms

2. **Added the following elements to ensure reproducibility:**

Complete search syntax with Boolean operators

Both controlled vocabulary (e.g., MeSH) and free-text terms

Number of records retrieved from each database

3. **Cited these supplementary tables in the Methods section (subsection "Search strategy", Page 5, Line 107).**

**Changes made:**

S2 Table and S3 Table: Provided complete search syntax for all databases.

**Comment 8:** Discussion Section --- Deficiencies in Comparison with Existing Studies: When discussing the model performance and predictive factors, there is a lack of sufficient comparison with similar studies, making it impossible to highlight the advantages and disadvantages of this study. It is recommended to supplement relevant comparative analysis to clarify the positioning of the study.

**Response:**

We have added a dedicated subsection (Page 21-22) comparing our findings with 4 key previous reviews[4-7]. The new analysis:

1) Quantifies performance evolution (AUC improvement trends)
2) Identifies paradigm shifts in predictor selection

**Changes made:**

Added comparative analysis: Page 21-22.

New citations supporting the comparison: References 59-62.

**Comment 9:** Clinical recommendations lack specific implementation paths: The proposed clinical practice recommendations are rather general, such as using AI and ML methods, conducting model validation, etc., and lack specific implementation steps and precautions. It is recommended to refine the recommendations to enhance their operability.

**Response:**

To refine clinical implementation recommendations, we have further specified: (1) the procedural steps for applying AI and machine learning methodologies, (2) concrete approaches for conducting external model validation, and (3) detailed strategies for clinical deployment through web-based calculators or applications, thereby ensuring operational feasibility and generalizability.

**Changes made:**

Replaced generic suggestions with actionable protocols: Page 22-23.

**Declaration:** All co-authors have approved the revised manuscript and our responses to the reviewers.

**References:**

[1].	Collins, G.S., et al., Transparent reporting of a multivariable prediction model for individual prognosis or diagnosis (TRIPOD): the TRIPOD statement. BMJ, 2015. 350: p. g7594.

[2].	Dreyer, N.A., et al., Why observational studies should be among the tools used in comparative effectiveness research. Health Aff (Millwood), 2010. 29(10): p. 1818-25.

[3].	Steyerberg, E.W., et al., Prognosis Research Strategy (PROGRESS) 3: prognostic model research. PLoS Med, 2013. 10(2): p. e1001381.

[4].	Beran, A., et al., Predictors of Post-endoscopic Retrograde Cholangiopancreatography Pancreatitis: A Comprehensive Systematic Review and Meta-analysis. Clinical Gastroenterology and Hepatology.

[5].	Sperna Weiland, C.J., et al., Preventive Measures and Risk Factors for Post-ERCP Pancreatitis: A Systematic Review and Individual Patient Data Meta-Analysis. Digestive Diseases and Sciences, 2024. 69(12): p. 4476-4488.

[6].	Sabrie, N., et al., Performance of Clinical Risk Prediction Models for Post-ERCP Pancreatitis: A Systematic Review. Pancreas, 9900.

[7].	Ding, X., F. Zhang and Y. Wang, Risk factors for post-ERCP pancreatitis: A systematic review and meta-analysis. Surgeon, 2015. 13(4): p. 218-29.
