# Supplementary material for: Risk prediction model for post-endoscopic retrograde cholangiopancreatography pancreatitis: A systematic review and meta-analysis
Source: PLoS One. 2025 Sep 15;20(9):e0332378. doi: 10.1371/journal.pone.0332378 (PMC12435719; doi:10.1371/journal.pone.0332378)
Supplement: Attachment — Submitted filename: Response to Reviewers_R2.doc [file pone.0332378.s015.doc]

Dear Dr. Suphakarn Techapongsatorn,

Thank you for your constructive feedback on our manuscript titled "Risk prediction model for post-endoscopic retrograde cholangiopancreatography pancreatitis: A systematic review and meta-analysis" (PONE-D-24-50343R1). We sincerely appreciate the time and effort invested by the academic editor and reviewers in evaluating our work.

We have carefully addressed all the comments and suggestions raised during the review process. Major revisions are highlighted in **yellow** in the manuscript. Below is a summary of the key revisions made:

**Reviewer #1**

The article has been revised very well. It has certain scientific significance, so I suggest it be published.

**Response:**

Thank you for your positive feedback on our revised manuscript. We greatly appreciate Reviewer 1’s acknowledgment of our revisions and the recommendation for publication. As suggested, we have thoroughly addressed all previous comments in the first round of revision, and we are delighted that the revised version meets the journal’s standards.

**Reviewer #2**

We sincerely thank the reviewer for the constructive and detailed feedback, which has significantly improved the clarity, methodological rigor, and clinical relevance of our manuscript. Below we provide a point-by-point response, with all changes incorporated into the revised version.

**1. Concern about the match between study purpose and the systematic review method**

**Reviewer’s comment:**

The author needs to deeply reflect and clarify whether the purpose of this study matches the method of systematic review. From the review of the current research situation, the author emphasized the importance of predictive models and proposed that there are differences in the quality of different models. Therefore, a systematic review is necessary. In fact, the factors included in predictive models vary in different studies, and the existence of differences is certain. Therefore, from the perspective that the systematic review aims to integrate the results of various studies, the author needs to further clarify the matching between the purpose and the methods when discussing the significance of the research.

**Response:**

We appreciate the reviewer’s observation. In the revised Introduction (**page 5-6, lines 100–126**) and Discussion (**page 27, lines 573–579**), we have explicitly clarified the methodological appropriateness of using a systematic review and meta-analysis for this topic. While heterogeneity in predictors is expected, such variability does not diminish the value of synthesis; rather, pooling allows us to (i) identify predictors with stable and clinically meaningful effects, (ii) quantify the predictive performance of models, and (iii) estimate the pooled incidence of PEP. This integrated approach provides a robust evidence base for refining models and guiding clinical application, thereby aligning the study purpose with the chosen methodology.

**2. Need for three core quantitative results**

**Reviewer’s comment:**

Based on the existing methodological framework, the authors need to incorporate the following three core results: ① Meta-analysis of the efficacy of predictive models; ② Meta-analysis of the incidence of PEP; ③ Meta-analysis results of each influencing factor.

**Response:**

We have fully incorporated these three core analyses into the Methods, Results, and associated figures/tables:

1.Meta-analysis of the incidence of PEP — We pooled data from 37 studies (n=38,016 patients) using a random-effects model with logit transformation, providing overall and subgroup estimates (**page 12-13, lines 245–277; Figure 3, Figure S1-5**).

2.Meta-analysis of individual predictors — For predictors reported in ≥3 studies, we extracted effect sizes (OR/RR/HR) and pooled them using random-effects models, identifying those with consistent, statistically significant associations (**page 13-14, lines 280–304; Table 3, Figure S6A-K**).

3.Meta-analysis of predictive model performance — We synthesized AUCs using random-effects and bivariate models, with results presented in forest plots (**page 14-17, lines 306–360; Figure 4, Figure S7A-H**).

Statistical methods for each analysis are detailed in the Statistical Analysis section (**page 9–10, lines 181-208**), including heterogeneity assessment, subgroup/sensitivity analyses, and publication bias evaluation.

**3. Structural clarity and content detail**

**Reviewer’s comment:**

Overall, the structure is chaotic, the content of each part is not detailed enough.

**Response:**

We have substantially restructured the manuscript to improve clarity and coherence:

- Introduction — Now explicitly outlines the clinical background, research gap, necessity of a systematic review, and alignment of aims with methods (**page 4–6, lines 88-126**).
- Results — Divided into three clearly labeled subsections corresponding to the three meta-analyses, each with summary statistics, heterogeneity measures, and visualizations (**page 12–17, lines 245-360**).
- Discussion — Reorganized into three principal findings, each aligned with a results subsection, followed by methodological implications, strengths/limitations, and clinical/research recommendations(**page 22–28, lines 470-603**).

All figures, tables, and supplementary materials are now cross-referenced in the text to improve navigability.

**4. Additional methodological rigor**

We followed PRISMA 2020 and PROBAST guidelines, and explicitly described data extraction, risk-of-bias assessment, and meta-analytic methods (including transformations, model choice, and effect size conversions). This ensures reproducibility and transparency in accordance with best practices for prediction model reviews.

**Additional Revisions:**Updated the PRISMA checklist (Table S1).Added a supplemental file with expanded data on incidence, risk factors, and predictive performance.We believe these revisions have significantly improved the manuscript and hope it now meets PLOS ONE’s publication standards. The revised files include:Response to Reviewers (this letter).Revised Manuscript with Track Changes (highlighting all edits).Manuscript (clean version).Should further modifications be required, we are happy to comply. Thank you again for your consideration.

Sincerely,

Yijun Mao, Qiang Liu, Hui Fan, Wenjing He, Cheng Zhang, Xueqian Ouyang, Erqing Li, Xiaojuan Wang, Li Qiu, Huanni Dong

Xianyang Central Hospital

[hlb33288602@163.com](mailto:hlb33288602@163.com)
